# Evaluation of candidate data-based welfare indicators for veal calves in Switzerland

**DOI:** 10.3389/fvets.2024.1436719

**Published:** 2024-07-17

**Authors:** Sibylle Zwygart, Barbara Lutz, Beat Thomann, Dimitri Stucki, Mireille Meylan, Jens Becker

**Affiliations:** ^1^Clinic for Ruminants, Vetsuisse Faculty, University of Bern, Bern, Switzerland; ^2^Centre for Proper Housing of Ruminants and Pigs, Federal Food Safety and Veterinary Office, Agroscope, Ettenhausen, Switzerland; ^3^Veterinary Public Health Institute, Vetsuisse Faculty, University of Bern, Bern, Switzerland

**Keywords:** fattening calves, bovine, digitalization, routine data, assessment

## Abstract

Welfare assessment protocols have been developed for dairy cows and veal calves during the past decades. One practical use of such protocols may be conducting welfare assessments by using routinely collected digital data (i.e., data-based assessment). This approach can allow for continuous monitoring of animal welfare in a large number of farms. It recognises changes in the animal welfare status over time and enables comparison between farms. Since no comprehensive data-based assessment for veal calves is currently available, the purposes of this review are (i) to provide an overview of single existing data-based indicators for veal calves and (ii) to work out the necessary requirements for data-based indicators to be used in a comprehensive welfare assessment for veal calves in Switzerland. We used the Welfare Quality Protocol^®^ (WQ) for veal calves and the Terrestrial Animal Health Code from the World Organisation of Animal Health for guidance throughout this process. Subsequently, routinely collected data were evaluated as data sources for welfare assessment in Swiss veal operations. The four WQ principles reflecting animal welfare, i.e., *‘good feeding’*, ‘*good housing’*, ‘*good health’* and *‘appropriate behaviour’* were scarcely reflected in routinely available data. Animal health, as one element of animal welfare, could be partially assessed using data-based indicators through evaluation of mortality, treatments, and carcass traits. No data-based indicators reflecting feeding, housing and animal behaviour were available. Thus, it is not possible to assess welfare in its multidimensionality using routinely collected digital data in Swiss veal calves to date. A major underlying difficulty is to differentiate between veal calves and other youngstock using routine data, since an identifying category for veal calves is missing in official Swiss databases. In order to infer animal welfare from routine data, adaptations of data collection strategies and animal identification are required. Data-based welfare assessment could then be used to complement on-farm assessments efficiently and, e.g., to attribute financial incentives for specifically high welfare standards accordingly.

## 1 Introduction

Products used to cover essential human needs originate partly from farm animals, including food, sanitary products, and clothing. Farm animal husbandry, however, has also been described to have detrimental effects on humans, for example through climate- and health-damaging emissions in industrial animal production systems, and impaired animal welfare has been reported repeatedly as a source of concern (1). In order to consume products of animal origin in a sustainable way in the future, a general restructuring of animal husbandry towards a more sustainable production with higher welfare standards will likely be needed. Higher welfare may be associated with lower mortality, lower antimicrobial use and a more efficient feed conversion. Whenever efforts are undertaken to restructure animal husbandry, factors indicative of animal welfare must be closely monitored to ensure acceptable or, ideally, gradually improving animal welfare standards in this process. According to the animal welfare legislation in the EU and Switzerland, it is mandatory to protect the life and well-being of animals (2–4).

Calves born on dairy farms considered surplus are often transported at a young age to specialised calf fattening farms, where they are fattened intensively for approximately 4–10 months, depending on local habits of the respective country (5–7). Transportation of calves has long been recognised as affecting health negatively (8–11). During transportation as well as during the fattening period, calves are frequently mixed with calves from other birth farms, exposing them to stress and pathogens. In addition, calves may be housed in barns with suboptimal barn climate and at a high stocking density. Management and housing characteristics are known to be important risk factors for either mortality or antimicrobial treatment (12, 13). In Switzerland, calves are frequently fattened in buildings which were initially intended for other purposes, such as unused pig barns (14). Also, in many cases, farms are not specialised on veal calf fattening but produce milk and veal alongside arable crops. Male veal calves, but also surplus female veal calves, are fed a high energy diet and slaughtered after a fattening period as veal calves (15). Veal is defined as meat originating from calves slaughtered at the age of up to 12 months in the European Union (16) and until a maximum carcass weight of 160 kg in Switzerland and a maximum of 8 months of age (17). The feed used is mainly fresh milk as well as milk replacer [produced from dehydrated milk, by-products of cheese making (e.g., whey), among others] dissolved in water (15, 18). In Switzerland, impaired welfare and high antimicrobial use call for restructuring of the current veal calf husbandry. High use of antimicrobials including highest priority critically important antimicrobials has been reported repeatedly in Swiss veal calf fattening operations (12, 13, 19–22). These treatments aim at controlling frequently occurring infectious diseases, predominantly diseases of the respiratory tract (12, 13). Antimicrobials may be injected into the body or administered via the feed, as metaphylaxis or as therapeutic treatment (12, 22).

As the definition of welfare is complex, multidimensional and dependent on societal values, it is not trivial to assess (23). To reflect the multidimensionality of welfare, a set of indicators must be used. These indicators can be categorised as resource-based, management-based, outcome-based or animal-based indicators (24, 25). Resource-based indicators (e.g., ‘*barn size’* or ‘*water provision’*) and management-based indicators (e.g., ‘*animal feeding’* and ‘*handling’*) describe the husbandry system and management, and its effects on animal welfare. However, these indicators do not measure animal features, and, therefore, can only partially reflect animal welfare. In contrast, outcome-based and animal-based indicators (e.g., ‘*slaughter carcass quality’* and ‘*physical appearance’* or ‘*stereotypic behaviour’*, respectively) are measured directly at the animal level and can thus describe actual animal welfare (26).

The World Organisation for Animal Health (WOAH), in its ‘Terrestrial Animal Health Code’ (TAHC) (27) proposes that welfare should be assessed using outcome-based and animal-based indicators. Over the past decades, several animal-based on-farm assessment protocols for a variety of farm animals have been developed. Examples are the Animal Needs Index (28), the ‘Board of Trustees for Technology and Buildings in Agriculture’ [Kuratorium für Technik und Bauwesen in der Landwirtschaft, KTBL, (29)], and the WelfareQuality® Protocol (WQ) (30), of which only the WQ also contains a welfare assessment protocol explicitly for veal calves, that must be conducted on-farm (30). A considerable disadvantage of on-farm assessments is, however, that they are not practical for assessments on a large number of farms because they are time-consuming. Furthermore, on-farm assessments are subject to observer bias even if observers are well trained and prepared (31). In addition, on-farm assessments provide only occasional insights and may not be used to describe variations of the welfare status of numerous farms over time. For this purpose, a methodology to assess welfare continuously and objectively over time is needed. Routine herd data, such as animal movement data, slaughter data or veterinary treatment data, are registered through official institutions and may be obtained simultaneously for many farms over an extended time period. By using this approach, welfare monitoring could be done in an increasingly objective way as well as in a time- and cost-efficient way.

For dairy cows, several publications report the assessment of welfare based on available herd data (32–35). The national surveillance program in the Netherlands uses digital data to monitor trends in cattle health, considered to be a part of welfare (36). For example, Santman-Berends et al. (37, 38) used the indicators ‘*mortality rate*’ and ‘*herds with cattle import’* (i.e., import from abroad). The use of routinely collected data has also been investigated for veal calves, with several studies using routinely collected data to analyse risk factors for disease (37, 39–41). None of the aforementioned investigations allowed for an exhaustive welfare assessment.

In the following, we review the availability and usability of routinely collected digital animal health data for Swiss veal calves to assess animal welfare. The review at hand was conducted within the framework of the Smart Animal Health project (42) and did not include Precision Livestock Farming (PLF) technologies, as corresponding reviews of commercially available and scientifically validated PLF technologies for health and welfare monitoring of livestock, and specifically for veal calves, have been reported separately (43, 44). We chose veal calves as the target production type as this production branch needs imminent restructuring due to high prevalence of disease, high antimicrobial use and high prevalence of resistant bacteria (12, 13, 19–21, 45–47). We used Swiss farms and available databases as well as the Swiss legislative framework as a model, yet assuming that many of our findings may be applicable in other countries as well. A combined section of results and discussion of our findings aims at providing guidance towards comprehensive, objective, and reliable data-based indicators for future welfare assessments in veal calf operations.

## 2 Materials and methods

This research project consisted of four main steps. In the first step, a systematic literature search was conducted, aiming at identifying existing data-based welfare indicators to assess welfare in Swiss veal calves. It was conducted in July 2019 and updated in May 2020 (last access on 2020-05-20) screening the following databases: PubMed, Web of Science, Scopus, CAB Direct, and Science direct (48–52). The search queries for the initial literature search were defined by a panel of 12 experts in the field (veterinarians, agronomists, animal welfare experts, researchers) (Table 1). Only peer-reviewed publications from 1995 onwards, written in English, French or German, were considered.

**Table 1:** Search terms for the systematic literature research to identify literature on data-based welfare indicators for veal calves.

	Search terms related to data, welfare and health		Synonyms for veal calves
Routine herd data	Routinely collected data OR routine herd data OR herd data OR census data OR pre-collected data OR national database OR register based	AND	Calf OR calves OR veal calf OR veal calves OR fattening OR fattened calf
Welfare	Welfare OR welfare assessment OR welfare quality OR well-being OR animal-based OR health OR health assessment OR health monitoring OR monitoring OR surveillance OR health indicator		
Others	Movement OR transport OR mortality OR carcass OR carcass condemnation		

The second step consisted of investigating which currently routinely collected data of existing Swiss databases is of practical use for generating data-based indicators informing welfare. To this end, we complemented the literature search with practical information from existing databases in the field, i.e., Swiss animal movement database (TVD) (53), meat inspection database (Fleischkontrolldatenbank, Fleko+) (54), and information system on antibiotics in veterinary medicine (Informationssystem für den Antibiotikaverbrauch in der Veterinärmedizin, IS ABV) (55).

Third, to improve integration in the production chain and to improve acceptance in the branch of industry, in February 2020, stakeholders were consulted to assess feasibility of the suggested data-based approach to assess welfare. A total of 11 stakeholders were involved (veterinarians, farmers, consumer organisations, animal protectionists, representants of the meat processing industry).

Finally, we brought together all findings, in order to report and discuss to what extent available data-based indicators describe welfare in its multidimensionality. In contrast to the query terms used for the literature search, we here used a list of proposed on-farm welfare indicators for guidance (i.e., non-data-based). On-farm welfare indicators were extracted from WQ protocol and TAHC (27, 30). We referred to the indicators within the WQ protocol for veal calves, as it is, to our knowledge, the only on-farm, animal-based welfare assessment currently available explicitly for veal calves (30). We also used indicators from the TAHC initially developed for dairy cows and beef cattle and checked them for practicability to be used in Swiss veal calves (27).

## 3 Results and discussion

The systematic literature review did not reveal any established data-based welfare indicators or comprehensive welfare assessment method available for veal calves using routinely collected data. In the following, we report and discuss the state of data-based welfare assessment for Swiss veal calves. The section is divided in two parts (general and specific observations and comments). Cited references indicate published information, whereas statements without cited reference are authors’ appraisals.

### 3.1 General observations regarding efficient data collection and processing

#### 3.1.1 Data availability

Usable animal welfare indicators must be available and reliable (56, 57). Data may be collected but not available, for example due to legal constraints, and treatment data cannot be retrieved without consent from both farmer and treating veterinarian. Taking slaughter carcass weight data as an example, reliability can only be assured if weight data is obtained from calibrated scales across all slaughterhouses for reproducibility purposes. Scales are relatively easy to use in practice everywhere, i.e., both in industrial slaughterhouses as well as in small butcheries. Other indicators, however, such as those related to carcass meat and fat share are assessed by human observers and can therefore be subject to bias within and among slaughterhouses (58). Furthermore, even if data are both available and reliable, they may not have been collected for the purpose of assessing animal welfare and may lead to inaccuracies for a welfare-related assessment (59). For instance, animal movement databases are meant to track locations the animal has been transported to (e.g., other farm(s), summer pasture, slaughterhouse), but disregard the time and distance spent on the road between locations. This missing information could be useful to determine welfare-related modalities of transport such as transport time. Furthermore, data structure determines if indicators can be calculated. For example, evaluating information from a free text field is complex as entries are not standardised (60). Free text data should be replaced or at least complemented with drop-down selections of pre-defined categories for easy processing. The use of standardised diagnosis codes would promote the harmonisation of information.

#### 3.1.2 Interpretability of indicators

Reliable welfare indicators should be adapted to the data available in the respective country, epidemiologically correct and understandable for involved people (61). This applies, for example, to the assessment of calf mortality, as it can be assessed using various populations as suggested by Santman-Berends et al. (61) (calves born alive, or already ear-tagged calves, or preweaned calves, among others). Available data may be retrieved from various origins, for instance, from a national government-run database, from a private database for a speciality branch of agriculture, or from the farmer’s notes, depending on the organisational structure of the country. The organisational level may entail or solve practical problems, especially important in multilingual countries like Switzerland, since regional or private databases may be available in specific languages only, thus hampering retrieving information or linking of databases.

Indicators are often used for classification, for instance, to delimit ‘acceptable’ from ‘not acceptable’. An objective way of assessment of what is acceptable is needed, for example, a maximum acceptable group size. Such threshold values may vary between label production programs (18, 62, 63).

#### 3.1.3 Multiple use of indicators

A further challenge for classification arises from the fact that some indicators can be related both to welfare concerns and also serve as indicators for other problems. For example, the indicators ‘mortality’ or ‘underdeveloped calves’, may either be used as a self-standing indicator, i.e., indicating the percentage individuals dying throughout the observation period and the percentage of underdeveloped calves in relation to the standard, respectively, or indicate poor husbandry if elevated, and inadequate feed supply if elevated, respectively.

#### 3.1.4 Merging data from different databases

In Switzerland, routine herd data are stored in various databases operated by governmental institutions or private organisations (53–55, 64). Content, structure, and availability of the data vary between databases. Data is provided in a way that suits the respective purpose; however, it has been found to be largely incompatible for merging among databases. For example, each veal calf has a unique identifier (ear tag number), which could be used for merging. In some databases, instead of using the complete identifier, shortened identifiers are used. This hampers merging information from various databases. A revision of national databases containing data on animals would be desirable in order to standardise terms and definitions and allow better merging.

#### 3.1.5 Distinguishing production types

In order to assess animal welfare for animals in a specific production type, such as veal calves, dairy calves, calves from cow-calf operations, heifers or dairy cows, it is necessary to correctly attribute individual animals to the respective production type. This is currently not possible using available routine data in Switzerland. Therefore, an alternative approach must be used to attribute animals to the corresponding production type, which is likely to be less precise. To filter veal calves from a cattle dataset, an approximate approach based on age, breed and sex can be used. We specifically encountered this problem in Switzerland, as providing this information is not mandatory for the farmer, and, if declared voluntarily, only two production types are available for cattle in the national animal database for bovines (‘dairy cows’ and ‘others’) (53). As a result, veal calves cannot be distinguished from calves in other bovine production types with certainty. Also, slaughter age cannot be used to identify the production type for veal calves, as occasionally dairy calves may also be slaughtered at an early age. Misclassification of individual animals may represent a major drawback of a purely data-based approach. In order to allow differentiation of the production type of ‘fattening calf’ in the future, the national databases should at least contain the information whether a farm keeps veal calves. An additional specification of the production type in the animal movement database would allow the identification and allocation of individual animals.

#### 3.1.6 Effects of herd size

Herd size must be considered for the calculation of data-based indicators, as the impact of a single animal is higher in smaller herds, especially when calculating proportions. This is applicable in Swiss veal calf fattening operations, as herd size varies considerably among farms, and some herds are relatively small (13). For example, small herd sizes must be accounted for using an adequate sample size, or indicators must be calculated in a standardised procedure such as for a defined number of animals in a defined number of observed farms.

### 3.2 Specific findings regarding specific candidate indicators

The following groups of indicators are discussed in the same order as presented in the TAHC (27). An overview of all indicators selected for this study is given in Table 2.

**Table 2:** Overview of welfare indicators from the WelfareQuality^®^ protocol for veal calves^1^, the proposed indicators in the Terrestrial animal health code^2^ and the data-based indicators available in Switzerland.

Measurables TAHC	Indicators from WQ for veal calves	Proposed indicators TAHC	Data-based indicators available in Switzerland
Behaviour	Social behaviours; other behaviours; abnormal behaviours; qualitative behaviour assessment	Decreased feed intake; increased respiratory rate; panting; stereotypic behaviours; aggressive behaviours; depressive behaviours; other abnormal behaviours	None
Morbidity rates	Claw lesions; joint lesions; bursae; bitten tail/ear; lameness; coughing; abnormal breathing; nasal discharge; ocular discharge; liquid manure; bloated rumen; dull calf; obviously sick calves	Morbidity rates including disease; lameness; post-procedural complication injury rate	Treatment records; AMU database; slaughterhouse data: (pre-slaughter inspection; meat inspection)
Mortality rate	Mortality rate	Mortality rate	Mortality rate
Changes in weight and body condition	Body condition	Poor body condition; significant weight loss	Significant weight loss (pre-slaughter inspection); underdeveloped calves at slaughter (carcass classification)
Physical appearance	Spots of hard skin; cleanliness of calves; dull calves; wet calves	Presence of ectoparasites; abnormal coat colour or texture; excessive soiling with faeces mud or dirt; dehydration; emaciation	Pre-slaughter inspection (severe emaciation; reduced general condition; skin damage; excessive soiling)
Handling responses	Avoidance distance; qualitative behaviour assessment	Chute or race exit speed; chute or race behaviour score; percentage of animals slipping or falling; percentage of animals moved with an electric goad; percentage of animals striking fences or gates; percentage of animals injured during handling; such as broken horns; broken legs; and lacerations; percentage of animals vocalising during restraint.	None
Complications due to routine procedure management	Absence of pain induced by management practices: tail docking	Post procedure infection and swelling; myiasis; mortality	None

1 Welfare Quality® Consortium. Welfare quality® assessment protocol for cattle. Lelystad, Netherlands: (2009).

2 WOAH. Terrestrial Animal Health Code. (2023). https://www.woah.org/en/what-we-do/standards/codes-and-manuals/terrestrial-code-online-access/.

#### 3.2.1 Behaviour

Behavioural assessment, for example assessing ‘*play behaviour’*, is part of a comprehensive welfare assessment. Normal behaviour can indicate good welfare (65). Abnormal behaviour patterns, such as ‘*tongue rolling’* or ‘*cross-sucking’* may indicate suboptimal (behavioural) welfare, potentially arising from suboptimal husbandry (66–68). Furthermore, some diseases may also lead to changes in behaviour like decreased feeding behaviour and absence of social interactions (69). In the WQ, social behaviours (‘*social licking’* and ‘*head bumping’*), other behaviours (‘*running’*, ‘*jumping*’ and ‘*frolic behaviour’*), abnormal behaviours (‘*tongue rolling’*) and a ‘*Qualitative Behaviour Assessment’* are measured (30). The following measures are suggested in the TAHC to assess the behaviour: ‘*decreased feed intake’*, ‘*increased respiratory rate’*, ‘*panting’*, ‘*stereotypic behaviours’*, ‘*aggressive behaviours’*, ‘d*epressive behaviours’* and ‘*other abnormal behaviours’* (27). It must be considered that social behaviour of calves changes over time and certain behaviours, for instance ‘*social licking’* evolve ‘between weeks 9 and 13’ (70). Therefore, the WQ recommends assessing behaviour at a defined standardised age of 13–16 weeks upon arrival in fattening units for all animals of the observed population (30).

A data-based assessment of behaviour is currently not possible due to the lack of routinely collected data in Switzerland. Precision livestock farming could provide data in the future; for instance, accelerometers could be used to gather information on the behaviour of calves (71–73). However, the majority of PLF technologies are not yet advanced enough to be applied on a large scale, primarily due to technical limitations, high costs, and the need for standardisation and data comparability across different systems (74, 75).

#### 3.2.2 Morbidity rates

Morbidity rates provide an insight into the welfare status of a herd (76–79). In the WQ, ‘*lameness’*, ‘*coughing’*, ‘*abnormal breathing’*, ‘*nasal discharge’*, ‘*ocular discharge’*, ‘*liquid manure’*, ‘*bloated rumen’*, ‘*dull calves’* and ‘*obviously sick calves’* are assessed (30). According to the TAHC, clinical indicators such as ‘*disease’*, ‘*lameness’*, ‘*post-procedural complication’*, and ‘*injury rates’*, as well as ‘*post-mortem pathology’* should be considered (27). To date, morbidity rates are not available in routine herd data in Switzerland, but data indicating morbidity could be provided through treatment records, antimicrobial use (AMU) and slaughterhouse data (see below).

#### 3.2.3 Treatment records

In Switzerland, for every treatment, farmers must record the following information: animal identification, commercial name and amount of given product, indication for treatment, treatment dates, withdrawal period, date from which foods deriving from treated animal(s) are again fit for human consumption, and the name of the person prescribing, delivering on stock or administering the therapeutic product (80). However, it is known that treatment journals are often incomplete (81). In Switzerland, treatments, when recorded, are mainly documented in an analogous form on paper (except for antimicrobial treatment data as described below). Data-based analysis of multiple farms, however, would require digital data recording, which is rarely done for treatments, other than for antimicrobial drugs in Switzerland.

Treatment data can only be a reliable indicator of morbidity if treatments are administered exclusively to diseased individuals, which is not the case if healthy individuals receive metaphylactic treatment, in veal calves mostly antimicrobial treatment (see below).

In contrast, sick animals do not necessarily receive treatment and individuals that never received treatment may nevertheless have experienced episodes of disease. Given that farmers usually do not have expert veterinary knowledge, they cannot be expected to distinguish sick from healthy individuals using the same approach as veterinarians or to establish accurate diagnoses for sick animals. In the light of these shortcomings, treatment data (and diagnostic data if available) must be interpreted with caution according to their source (e.g., records from veterinarians or from farmers). Thus, with the currently available data from farmers’ treatment journals in Swiss veal farms, an accurate estimate of morbidity rates (80) is not possible.

#### 3.2.4 Antimicrobial use and treatment incidence

A further possibility is to use antimicrobial use (AMU) as an indicator of morbidity rate. In Swiss veal calves, antimicrobial treatments have been reported to account for 21–30 daily doses per calf-year (12, 82). Also, Swiss veal calves are administered by far the largest share of critically important antimicrobials administered to livestock (83). In Switzerland, veterinarians are obliged by law to document AMU (date, number of animals, indication, name of commercial product administered, application days, amount per animal and application, total amount, delivered amount to farmer, application route) in a government-driven AMU-database (55). Veterinarians are not obliged to document AMU at the animal level. The Swiss legislation allows the delivery of on-stock supplies of specific antimicrobials to farmers by veterinarians as long as the veterinarian is familiar with the farm and the animals to be treated and has a corresponding contract with the farmer (Art. 7c 2) (80). Such antimicrobials are not administered immediately to animals, and only limited information, e.g., the animal species the antimicrobial will be used for, is specified in the AMU-database for on-stock supplies, but neither a production type nor an indication for treatment. Approximatively 25% of registered antimicrobial prescriptions were prescriptions of antimicrobials supplied on-stock in 2022 in Switzerland (83). Consequently, AMU must be used with caution as an indicator of animal health, as antimicrobial treatments may be wrongly attributed for various reasons (not yet administered on-stock drugs, unused antimicrobial beyond expiration date, antimicrobial not used because flask was broken, among others).

On the other hand, calves estimated to be at risk of disease often undergo metaphylactic antimicrobial group treatments in Swiss veal operations (6, 22, 84). This way, healthy individuals receive antimicrobials in a group alongside diseased individuals, therefore the amount of drug used may be only weakly, or not at all, correlated with disease incidence.

Another reason to use AMU with caution as a health indicator is the fact that false incentives may emerge. Considering low AMU as an indicator of good welfare may represent an incentive to leave diseased calves untreated. To prevent this, AMU as an indicator should be used in combination with other parameters, e.g., mortality (39, 85) or data from meat inspection, in order to interpret AMU data in the light of the best possible evaluation of the calves’ health status. On the contrary, more antimicrobials than necessary may be used in other farms based on individual management decisions (e.g., intensive metaphylactic treatments upon arrival of the calves in the fattening facility) although animal welfare would be acceptable. Thus, high AMU must not reflect frequent morbidities under such conditions.

To obtain quantitative information about AMU that is comparable, e.g., among farms and among regions or countries, appropriate calculations to standardise treatment incidence assessment must be considered. The European Medicines Agency (EMA) proposes the defined daily dose (DDD) for a standardised comparison of national AMU (86, 87). Defined daily doses are the respective doses of each antimicrobial drug needed to correctly treat 1 kg of live animal weight for the main indication for 1 day. The EMA DDD values were shown to be applicable for therapeutic products licenced in Switzerland (88). As an alternative to DDD’s, Kasabova et al. (89) suggest applying a similar measure (used daily dose, UDD) for benchmarking of farms, as UDD may be more precise, with less under- or overestimation than DDD. For both approaches, more detailed raw treatment data than the amount of AMU provided by the veterinarian for a farm (possibly including on-stock supplies) is needed, i.e., the number of treated animals, the dosage used in mg/kg and the treatment duration. Thus, it may be possible to estimate AMU if raw data is of excellent quality, however, its suitability as a self-standing indicator of animal welfare is questionable, as AMU is not necessarily correlated with morbidities.

#### 3.2.5 Animal welfare monitoring at the slaughterhouse

Another possibility to collect data on morbidities in calves is the use of slaughterhouse data. An important aim of meat inspection (MI) is to discard carcasses which are unfit for human consumption (90). To date, MI has also become important in monitoring animal welfare. According to Grandin (91), there is great potential in monitoring animal welfare at the slaughterhouse. For example, ‘*body condition score’*, ‘*lesions*, *injuries’*, ‘*lameness’*, ‘*cleanliness’*, or *‘pathological changes in the organs’* of the animals could give an insight into the conditions on a farm. In Switzerland, cull animals must undergo an ante-mortem inspection, which includes, among others, assessment of the general condition of the animal and of specific signs of disease (Art. 4^29^) (92). During ante-mortem inspection, it is possible to detect some welfare issues on the farm, such as pronounced weight loss or excessive soiling (93). After slaughter, the carcass and internal organs are examined for pathological changes (Art. 30) (92). Carcasses that are found to be unfit for human consumption are condemned, whereby a distinction is made between whole carcass condemnation (WCC) and partial carcass condemnation (PCC). Although WCC is less common in calves than in cows or pigs, its frequency can provide useful information, as such condemnations may be indicative of severe lesions or pronounced weight loss (94). Partial carcass condemnation, where only organs or parts of the carcass are condemned (95, 96) can provide information about the health of the animals before slaughter. Its frequency must, however, also be interpreted with caution, as it has been shown that clinical symptoms do not necessarily correspond to the findings at the slaughterhouse (45). For example, not all calves with pathological lung alterations upon slaughter could be identified during clinical on-farm examination before slaughter (45, 97, 98).

As the primary purpose of MI is to ensure food safety for humans, not all information relevant for assessing animal welfare are collected. For example, organs relevant for food safety (e.g., kidneys, liver, lungs, lymph nodes) are examined, whereas examination of the abomasum, which could provide evidence of abomasal ulceration known to be associated with stress and thus welfare issues in veal calves, is omitted. Correspondingly, not every pathological lesion (potentially negatively associated with welfare) can be identified upon MI. In addition, time pressure at the slaughter line may reduce recording quality (94). Thus, WCC and PCC data should be used with care for any other purpose than food safety (58).

Furthermore, recording procedures may vary among slaughterhouses, so the effect of the slaughterhouse must be considered as well (58). Varying procedures and thus varying data quality may arise from specific working conditions in slaughterhouses or requirement of specific customers (58). In contrast, WCC due to injuries and weight loss were mostly recorded at smaller slaughterhouses in Switzerland, as larger ones reject such animals upon arrival in the first place (41). To include a dataset as exhaustive as possible for evaluation of animal welfare, data from both small and large slaughterhouses must be included (41). In summary, as AMU and treatment data can provide limited information about morbidity rates on the farm, slaughterhouse data can only reveal particularly serious problems on farms. Nevertheless, the actual morbidity rates cannot be correctly determined with the data currently available in Switzerland.

#### 3.2.6 Mortality rates

In both WQ and TAHC, ‘*mortality rate’* is used as a welfare indicator (27, 30). Mortality either indicates animal welfare problems directly as animals may have suffered before death or euthanasia, or mortality is used as an indicator of different surrounding issues (e.g., management issues, or poor housing conditions). In Switzerland, succumbed calves must be reported to an animal movement database, thus mortality rates can be calculated using these data. Mortality rates can be calculated in different ways and different time spans for reporting can be considered. While brief time spans from days to trimesters are considered for surveillance, a one-year period is usually chosen for welfare assessment (29, 30). To avoid overestimating the impact of a single calf on a herd’s mortality rate in small herds, it may be useful to extend the reporting period (99).

In addition, different calculation methods can be considered. For instance, a mortality rate calculated as dead calves per year / total calves ear-tagged per year may not take early movements of calves to another farm into account. The number of calves in the denominator may wrongly include calves that are no longer at risk of death on the farm, which artificially decreases mortality rates. An epidemiologically more accurate calculation would be to use days at risk in the denominator (the sum of all days all calves spend alive on the respective farm during the period of assessment).

Different age categories may also be considered when assessing mortality rates (61). As the farm of origin has an impact on the mortality of veal calves in fattening units (100), it may be useful to differentiate between mortality early after arrival on the fattening unit and mortality later in the fattening period. Mortality rates can be derived from routine herd data in Switzerland. However, determining the appropriate cut-off points necessitates further investigation in subsequent studies.

#### 3.2.7 Changes in body weight and condition

Good health is a prerequisite for a good growth performance, but growth rates can also be influenced by a multitude of other factors such as purchase of calves, management and feeding, among others (13, 101). Poor growth is either a welfare problem by itself, or it can indicate other problems like lack of colostrum supply, episodes of diarrhoea or respiratory disease, and bad housing and climatic conditions (29). In the WQ ‘*the percentage of calves that have a significantly lower body condition and weight than the average of the batch’* is assessed (30). The WOAH recommends in the TAHC to use ‘*poor body condition’* and ‘*significant weight loss’* as an indicator for compromised welfare (27).

There are two possibilities to identify calves with poor growth using routinely available digital Swiss herd data. One could be the use of carcass classification. Carcasses in Switzerland are routinely classified according to the CH TAX system, which includes estimations of meat share and fat share (102). Low meat share and low fat share are indicators for a poor body condition before slaughter, which, in turn, can indicate poor feeding or disease (103). Thus, it can be assumed that undeveloped calves usually also have lower carcass classification (104). It may be used as self-standing welfare indicator, as it directly provides information about growth performance. However, only few Swiss slaughterhouses currently transmit CH TAX classification data to the animal movement database, thus these data are not reliably available. Similarly, hot carcass weight data is only transmitted to that database to a similar extent.

Another possibility is to detect underdeveloped calves by measuring average daily weight gain. Calculating average daily gain (ADG, i.e., the mean daily live body weight gain per day over a given period) can be done in veal calves (47, 82). A data-based estimation of the ADG could be realised by using a standardised birth weight, the hot carcass weight, and the age at slaughter. However, instead of using a standardised birth weight, estimation of ADG would be more precise if actual birth weight or bodyweight at arrival at the fattening facility was used. In practice, this information is rarely available. Comparing ADG between farms is challenging, as feeding frequency and fattening intensity may differ. Moreover, some actors of the meat industry process calves as veal at a higher live body weight than usual in Switzerland (105). An ADG threshold (minimal daily weight gain) may be used to detect obviously sick calves, as underdeveloped calves are more likely to be in poor health. The percentage of underdeveloped calves could potentially be determined by combining age at slaughter, carcass weight, ADG, and carcass classification. To determine the corresponding methodology, further investigations are warranted.

To summarise, it may be possible to identify calves showing poor growth through data-based indicators with a combination of age at slaughter, slaughter weight, and carcass classification, but further research is needed to evaluate which combinations and cut-offs would be most appropriate. Correspondingly, data-based identification of calves showing poor growth can only be done after slaughter, as routinely measuring body weight and body condition in live calves is not feasible to date.

#### 3.2.8 Physical appearance (in live calves)

The physical appearance of a calf may provide information on management, health, and behaviour of its group mates. The following measures are assessed in the WQ: *‘spots of hard skin’*, *‘cleanliness of calves’*, *‘dull calves’* and *‘wet calves’* (30). The WOAH recommends in the TAHC to monitor the following measures: ‘*presence of ectoparasites’*, *‘abnormal coat colour’*, *‘abnormal coat texture’*, *‘excessive soiling with faeces, mud or dirt’*, *‘dehydration’* and *‘severe emaciation’* (27).

During ante-mortem examination at the slaughterhouse, severe emaciation, reduced general condition, skin damage, and excessive soiling can be recorded. These data could be used to identify farms with animal welfare concerns or an above average rate of serious health problems. However, as not all relevant pathologies are detected during ante-mortem inspection or MI, it is not possible to assess the actual physical appearance based on available routine herd data. Also, when performing ante-mortem inspection at the slaughterhouse, findings cannot be attributed to the farm with certainty, since above-mentioned conditions may have developed during transport. Physical appearance can thus only be captured to a minimal extent, and exclusively at the culmination of the fattening phase within the abattoir. While ongoing monitoring directly on the farm would be preferable, it remains a technical impossibility at present.

#### 3.2.9 Handling responses

Poor handling of animals may lead to impaired welfare as animals experience aversive emotions (106). In contrast, gentle handling of animals can reduce stress, as the animals have little negative experience with humans (107, 108). In the WQ, ‘*avoidance distance*’ is assessed to reflect the human-animal relationship (30). The WOAH recommends in the TAHC to record the following measurements: ‘*chute or race exit speed*’, ‘*chute or race behaviour score*’, ‘*percentage of animals slipping or falling*’, ‘*percentage of animals moved with an electric goad*’, ‘*percentage of animals striking fences or gates*’, ‘*percentage of animals injured during handling* (broken horns; broken legs and lacerations)’ and ‘*percentage of animals vocalizing during restraint*’ (27). Handling response, like other behavioural measurements, cannot be obtained with the data currently available for veal calves in Switzerland.

#### 3.2.10 Complications due to routine management practices

Routine management practices like tail docking, disbudding and castration are performed to improve animal performance, facilitate management, and improve human safety and animal welfare (27). The WQ assesses whether the procedure is performed under local anaesthesia and using appropriate analgesia (30). In the TAHC it is recommended to record ‘*infection*‘, ‘*swelling*’, ‘*myiasis*’ and ‘*mortality*’ due to routine procedures (27). In Switzerland, routine procedures are not performed on veal calves because they are usually slaughtered at an age of approximatively 160 days when horns are still very short, thus disbudding is not performed, and there is also no need for castration. Tail docking is prohibited in Switzerland. Hence, no data-based indicators for complications due to routine procedures were included in this review.

#### 3.2.11 Interim conlusion regarding specific candiadate indicators

This evaluation for veal calves is in line with the results of research done on data-based welfare assessment for dairy cows. In Scandinavian countries, although data quality is significantly better, no reliable statement could be made about the actual welfare status of dairy cows (34). Furthermore, data-based indicators for behavioural indicators are similarly lacking for dairy cows (32–35). On-farm assessment is still recommended for dairy cows (109), and currently, there is no possibility to infer veal calf welfare from digitally available data in Switzerland.

## 4 Conclusion

The data-based indicators proposed in the frame of this review partially describe multidimensional welfare. Suggested data-based indicators predominantly describe animal health (*‘morbidity rates’*, *‘mortality rates’*, *‘changes in weight and body condition’*, *‘physical appearance’*). Data-based indicators on behaviour are lacking. These indicators should therefore rather be called health indicators than welfare indicators. However, a comprehensive data-based assessment of animal health is currently not possible either. Morbidity rates cannot be fully captured with the available data, and physical appearance and changes in weight and body condition are not practical to be recorded directly on the farm. To date, only severe cases of impaired welfare may be detected at the slaughterhouse through data-based assessment. Before the proposed data-based indicators can be used, however, these indicators must be tested for applicability, reliability, and validity. Once testing and refinement have been concluded, and the respective databases provide the required quality for an accurate calculation, we are convinced that these indicators will provide a useful complementation to routine on-farm assessments.

## Data availability statement

The original contributions presented in the study are included in the article/supplementary material, further inquiries can be directed to the corresponding author.

## Author contributions

SZ: Writing – original draft, Writing – review & editing, Conceptualization, Data curation, Formal analysis, Investigation, Methodology, Visualization. BL: Writing – review & editing. BT: Writing – review & editing, Conceptualization, Methodology, Project administration. DS: Formal analysis, Methodology, Writing – review & editing. MM: Funding acquisition, Methodology, Resources, Supervision, Writing – original draft, Writing – review & editing. JB: Supervision, Writing – original draft, Writing – review & editing.
